# Amrubicin for relapsed small-cell lung cancer: a systematic review and meta-analysis of 803 patients

**DOI:** 10.1038/srep18999

**Published:** 2016-01-11

**Authors:** Nobuyuki Horita, Masaki Yamamoto, Takashi Sato, Toshinori Tsukahara, Hideyuki Nagakura, Ken Tashiro, Yuji Shibata, Hiroki Watanabe, Kenjiro Nagai, Kentaro Nakashima, Ryota Ushio, Misako Ikeda, Nobuaki Kobayashi, Masaharu Shinkai, Makoto Kudo, Takeshi Kaneko

**Affiliations:** 1 Department of Pulmonology, Yokohama City University Graduate School of Medicine, Yokohama, Japan; 2 Department of Pulmonology, Yokohama Minami Kyosai Hospital, Yokohama, Japan; 3 Respiratory Disease Center, Yokohama City University Medical Center, Yokohama, Japan

## Abstract

Currently, amrubicin is permitted for relapsed small-cell lung carcinoma (SCLC) only in Japan. The efficacy and adverse effects of amrubicin as reported by previous studies varied greatly. The inclusion criterion was a prospective study that was able to provide data for efficacy and safety by the AMR single agent regimen as second-line chemotherapy for a patient with SCLC. Binary data were meta-analyzed with the random-model generic inverse variance method. We included nine articles consisted of 803 patients. The pooled three-, six-, and nine-month progression-free survival were 63% (95% CI 57–69%, I^2^ = 53%), 28% (95% CI 21–35%, I^2^ = 71%), and 10% (95% CI 6–14%, I^2^ = 41%), respectively. The pooled six-, 12-, and 18-month overall survival were 69% (95% CI 61–78%, I^2^ = 83%), 36% (95% CI 28–44%, I^2^ = 80%), and 15% (95% CI 8–21%, I^2^ = 81%), respectively. Amrubicin seemed much more beneficial for Japanese patients. However, compared to the efficacy of topotecan presented in a previous meta-analysis, amrubicin may be a better treatment option than topotecan for both Japanese and Euro-American. Adverse effects by amrubicin were almost exclusively observed to be hematological. Notably, grade III/IV neutropenia incidence was 70% and febrile neutropenia incidence was 12%.

Small-cell lung carcinoma (SCLC) represents approximately 15% of all cases of lung cancer^1^. This cancer is characterized by rapid progression and a tendency to disseminate. Therefore, the majority of the patients are diagnosed after the disease has already progressed. Although SCLC is considered highly chemotherapy-sensitive, it usually relapses months later. As such, safe and effective second- or later-line chemotherapy regimens are required^2^,^3^. Currently, the topotecan (TOP) single agent regimen is the only regimen that has been proved to prolong overall survival (OS) of relapsed SCLC compared to the best supportive care^4^. This is why TOP is the most widely used chemotherapy regimen for relapsed or refractory SCLC^5^,^6^. However, it is known that TOP is not so effective for refractory-relapsed SCLC cases, who have relapsed within 60 or 90 days after the end of the previous, usually platinum doublet, chemotherapy. The objective response rate (RR) by TOP for refractory-relapsed cases has been estimated to be only 5%^7^.

Another promising chemotherapy regimen for relapsed SCLC is the amrubicin (AMR) single agent regimen^2^,^3^. AMR is a synthetic anthracycline with a structure similar to doxorubicin, which was approved in 2002 by the Japanese Government. Some previous studies suggested that AMR would be a good choice to treat relapsed SCLC, especially for refractory-relapsed cases and the Asian population^8^,^9^,^10^,^11^,^12^,^13^,^14^,^15^,^16^. The efficacy and safety of AMR are of considerable interest for all physicians who take care of patients with SCLC. However, the efficacy and adverse effect (AE) rate of AMR as reported by previous studies have seemed to vary greatly. Therefore, we tried to perform a systematic review and meta-analysis to provide data concerning objective response, survival, and AEs of AMR when prescribed as the second- or third-line chemotherapy for patients with SCLC.

## Methods

Institutional review board approval and patient consent were waivered because of the review nature of this study.

### Study search

Two investigators (H.N., N.K.) systematically searched eligible articles independently. This search was conducted using the PubMed, Web of Science, and Cochrane databases as of August 1^st^, 2015. The following search formula was used for PubMed: (“small-cell lung cancer” OR “small-cell lung carcinoma” OR “SCLC”) AND (relapsed OR refractory OR 2nd-line OR “second line” OR 3rd-line OR “third line” OR “previously treated”) AND (amrubicin OR AMR OR Calsed OR SM-5887). The inclusion criterion for a study to be included in this meta-analysis was that it should be a prospective study that was able to provide data for at least one of following outcomes by the AMR single agent regimen as second-line chemotherapy for a patient with SCLC: objective response, progression-free survival (PFS), overall survival (OS), hematological AE, non-hematological AE, and treatment-related death (TRD). Some cases in a study could be treated as third-line chemotherapy, however, if all cases were treated as third- or later-line chemotherapy, or if any case was treated as fourth- or later-line chemotherapy, the study was excluded. Eastern Cooperative Oncology Group performance status had to be two or better. A regimen should be considered to meet current standards: 30–45 mg/m^2^ on day 1–3. Thus, non-standard regimens used in phase I study and a non-standard weekly regimen were excluded. The report had to be written in English language as a full article. Duplicate uses of the same data were excluded.

### Outcome

For objective response analysis, we evaluated response rate (RR) and disease control rate (DCR). If neither the number nor rate of cases who satisfied objective response and disease control were directly provided, we calculated them as follows: RR was the sum of complete response and partial response, DCR was the sum of complete response, partial response, and stable disease^17^.

Three-month, six-month, nine-month PFS, and six-month, 12-month, and 18-month OS were evaluated. If necessary, Parmar’s method was used to estimate survival rate^18^.

The rate of AEs evaluated with National Cancer Institute-common toxicity criteria grade III or more were evaluated. Hematological toxicity such as neutropenia, thrombopenia, anemia, and febrile neutropenia; non-hematological toxicity including fatigue, nausea/vomiting, and cardiotoxicity; and TRD were evaluated. AEs were analyzed based on the number of patients, not based on the number of chemotherapy courses. The rate of AEs was evaluated using a per-protocol analysis, but not using an intention-to-treat analysis. This meant that patients who was assigned to an AMR arm but did not receive AMR were excluded from AE analysis.

### Statistics

We used the random-model generic inverse variance method^19^. Preceding the meta-analysis, the standard error was estimated with the Agrestia-Coullb method, as we could not obtain standard error for outcomes with prevalence of 0% by the commonly-used method (standard error = standard deviation/square-root of n)^20^. The heterogeneity evaluated with the I^2^ statistics was interpreted as follows: I^2^ = 0% indicated no heterogeneity, 0% < I^2^ < 25% indicated the least heterogeneity, 25% ≤ I^2^ < 50% indicated mild heterogeneity, 50% ≤ I^2^ < 75% indicated moderate heterogeneity, and 75% ≤ I^2^ indicating strong heterogeneity^21^. Because AMR had been approved only in Japan and because most studies were reported from Japan, we made two subgroups: reports from Japan and those not from Japan. The later sub-group was named the Euro-American group in this study, because non-Japanese studies were only from the USA and Germany, as described in the results section.

We used meta-regression to estimate objective response and survival for each of the sensitive/refractory relapse and Japanese/Euro-American settings in the second-line scenarios^19^,^22^. For meta-regression, we used “the sensitive/refractory relapse”, “Japanese/Euro-American settings”, and “second/third line treatment” as modulators. We obtained the data of responses, survivals, and adverse effects as interceptions at modulator = 0% or 100%.

Publication bias was evaluated through visual inspection of funnel plots^19^. We were planned to conduct Begg’s test to assess asymmetry of funnel plots when the number of included studies exceed 10^19^. However, this test was not conducted due to limited number of trials.

Random-effect model meta-analysis was performed using Reviewing Manager ver. 5.3 (Cochrane Collaboration, Oxford, UK)^19^. Mixed-model meta-regression was performed using “metafor” package on free software R^23^.

## Results

### Study search

**Figure 1:**
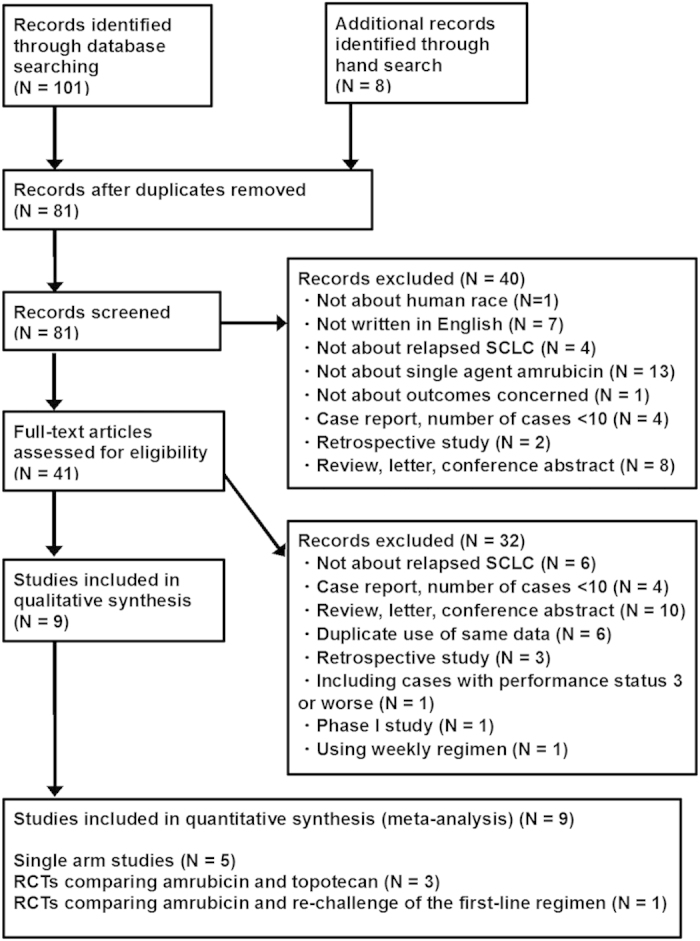
Flow chart for study search (PRISMA diagram).

Of 108 articles that met the preliminary criteria, we found nine eligible articles, which included five single arm studies, three RCTs that compared AMR and TOP, and an RCT that compared AMR and a re-challenge of the first-line chemotherapy (Fig. 1, Table 1)^8^,^9^,^10^,^11^,^12^,^13^,^14^,^15^,^16^. Six, two, and one study were reported from Japan, the USA, and Germany, respectively. Three studies from the USA and German were grouped as Euro-American studies. The number of SCLC patients in a study who were treated with AMR ranged from 27 to 424 with a median of 50. The total number of patients in all the studies was 803. In most of the studies, patients with performance status of one and male sex were the majority. The median age presented for each study ranged from 62 to 70 years. Two studies recruited only sensitive-relapse cases, two studies recruited only refractory-relapse cases, the other recruited both relapses. The cutoff between refractory and sensitive relapse was 90 days except for two studies that used 60 days and eight weeks. Five studies recruited only cases who were treated with AMR as second-line chemotherapy. The other four studies recruited cases receiving both second-line and third-line chemotherapy (Table 1). Eventually, 758 second-line cases (94.4%) and 45 third-line cases (5.6%) were included. Eight studies used an AMR dosage of 40 mg/m^2^ and one used 35 mg/m^2^.

### Objective response

**Figure 2:**
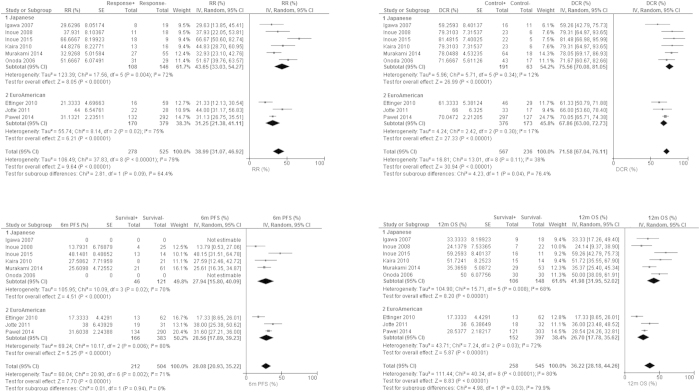
Forrest plots for objective responses and survival. RR: response rate. DCR: disease control rate. 6 m PFS: 6-months progression-free survival. 12 m OS: 12-months overall survival. SE: standard error. IV: inverse variance method. 95% CI: 95% confidence interval.

We evaluated objective responses from 803 cases belonging to nine studies (Figure 2, Supplementary Table 1). The pooled RR and DCR were 39% (95% confidence interval (CI) 31–47%, I^2^ = 79%) and 72% (95% CI 67–76%, I^2^ = 38%), respectively.

### Progression-free survival

Seven studies consisted of 716 patients provided data for PFS (Figure 2, Supplementary Table 1, Supplementary Figure 1). The pooled three-, six-, and nine-month PFS were 63% (95% CI 57–69%, I^2^ = 53%), 28% (95% CI 21–35%, I^2^ = 71%), 10% (95% CI 6–14%, I^2^ = 41%), respectively.

### Overall survival

All of nine studies presented data concerning OS (Figure 2, Supplementary Table 1, Supplementary Figure 1). The pooled six-, 12-, and 18-month OS were 69% (95% CI 61–78%, I^2^ = 83%), 36% (95% CI 28–44%, I^2^ = 80%), 15% (95% CI 8–21%, I^2^ = 81%), respectively (Figure 2).

### Meta-regression

We conducted the meta-regressions to estimate the responses and survival rates for four scenarios: sensitive-relapsed Japanese, refractory-relapsed Japanese, sensitive-relapsed Euro-American, and refractory-relapsed Euro-American. Although statistical significance was not constantly observed, sensitive-relapsed cases and cases in the Japanese study had better objective response and survivals (Supplementary Table 2). Even in Euro-American scenarios, AMR could lead to a reasonable objective response and survival (Table 2). For the sensitive-relapsed Euro-American scenario, RR, DCR, and 12-month OS were 43%, 69%, and 35% respectively. For the refractory-relapsed Euro-American scenario, RR, DCR, and 12-month OS were 19%, 68%, and 19% respectively.

### Adverse events

**Figure 3:**
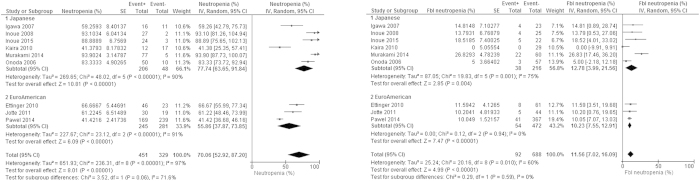
Forrest plots for adverse effects. Fbl neutropenia: febrile neutropenia. SE: standard error. IV: inverse variance method. 95% CI: 95% confidence interval.

We evaluated AEs from 803 cases belonging to nine studies (Figure 3, Supplementary Table 1, Supplementary Figure 1). The pooled hematological AE rates were 70% (95% CI 53–87%, I^2^ = 97%) for neutropenia, 24% (95% CI 17–30%, I^2^ = 75%) for thrombopenia, 19% (95% CI 13–26%, I^2^ = 76%) for anemia, and 12% (95% CI 7–16%, I^2^ = 60%) for febrile neutropenia. The pooled non-hematological AE rates were 2% (95% CI 0–5%, I^2^ = 0%) for nausea/vomitting, 7% (95% CI 2–12%, I^2^ = 76%) for fatigue, and 3% (95% CI 2–5%, I^2^ = 0%) for cardiotoxicity. TRD was observed in 1% (95% CI 0–4%, I^2^ = 0%).

### Publication bias assessment

No statistical test was conducted for publication bias because number of trials did not exceed 10. Visual inspection of funnel plots revealed no publication bias for any of the meta-analyses above (Supplementary Figure 2).

## Discussion

The efficacy and safety of AMR as second-line chemotherapy for SCLC has been evaluated in many phase II studies since 2006 (Table 1)^8^,^9^,^10^,^11^,^12^,^13^,^14^,^15^. Given the favorable results, von Pawel *et al.* recently conducted a phase III RCT to compare AMR and the current standard second-line treatment, the TOP single agent regimen^16^. These studies suggested that AMR can provide promising outcomes even for refractory-relapsed cases (Figure 2, Table 2, Supplementary Table 2).

In the sensitive-relapsed Euro-American scenario, AMR can bring RR of 43% and DCR of 69% (Table 2). These objective responses were much higher than the RR of 17% and DCR of 42% by TOP for sensitive-relapsed SCLC according to a recently published meta-analysis^7^. However, AMR could not greatly improve OS for sensitive-relapsed cases compared to TOP (Table 2)^7^. That is, this apparent high objective response by AMR does not correctly surrogate good OS for sensitive-relapsed cases.

In contrast, AMR was actually related to better OS than TOP for refractory-relapsed cases. In the refractory-relapsed Euro-American scenario, RR, DCR, six-month OS, and 12-month OS by AMR were 19%, 68%, 53%, and 19%, respectively (Table 2). According to the recent meta-analysis, RR, DCR, six-month OS, and 12-month OS by TOP were 5%, 29%, 37%, and 9%, respectively^7^. AMR seems a better treatment option for refractory-relapsed SCLC. Sub-analysis of only a phase III head-to-head trial comparing AMR and TOP also demonstrated a statistically significant OS benefit in favor of AMR compared to TOP (6.2 months versus 5.7 months; hazard ratio 0.766, *p* = 0.047)^16^.

Responses and survivals by AMR for SCLC were better in Japanese scenarios. For sensitive and refractory scenarios, 12-month OS were 51% and 34%, respectively (Table 2). We do not have clear explanation why there was the observed difference of benefit from AMR between Japanese and Euro-American. The Japanese guidelines emphasize the marginally positive statistical significance for OS in subgroup analysis detected in the phase III trial by Pawel *et al.* (*p* = 0.047)^24^, while National Comprehensive Cancer Network guidelines ignore this subgroup analysis^6^. Currently, only the Japanese guidelines recommend the use of AMR for refractory relapsed SCLC^6^,^24^. Thus Japanese researchers and oncologists were familiar with administrating AMR for relapsed SCLC^9^,^10^,^11^,^13^,^14^,^15^.

The safety profile of AMR is generally similar to that of TOP. AEs by AMR were almost exclusively observed to be hematological. Grade III/IV neutropenia was observed 70% of cases, which resulted in febrile neutropenia incidence of 12% (Figure 3).

Most guidelines recommend prophylactic granulocyte-colony stimulating factor for febrile neutropenia high-risk patients when administrating a chemotherapy regimen that can improve OS in compensation for an intermediate, 10–20%, risk for febrile neutropenia^25^,^26^,^27^. Advanced cancer, history of previous chemotherapy, age ≥65 years are usually counted as indicating a high risk. Therefore, prophylactic granulocyte-colony stimulating factor administration for patients who were treated by AMR for relapsed SCLC is usually justified. Other hematological AEs, thrombopenia and anemia, are usually treatable with blood transfusion. Although anthracycline-induced cardiotoxicity was often emphasized^28^, it was observed in only 3% of cases despite the elderly distribution of SCLC patients (Supplementary Figure 1). TRD was also rare (Supplementary Figure 1).

The current study has a few limitations. First, the number of included studies was not so large. However, we successfully derived pooled outcomes adjusting baseline patient characteristics using meta-regression. Second, we could find only three trials that directly compared AMR and TOP. As of 2015, AMR is only approved in Japan; however, this medication would potentially prolong the OS of refractory-relapsed Euro-American SCLC patients. Therefore, we hope there will be further trials comparing AMR and TOP in refractory-relapsed SCLC.

In conclusion, we conducted a systematic review and meta-analysis to evaluate objective response, survival, and AEs of AMR when prescribed for patients with relapsed SCLC. According to our analysis, compared to TOP, AMR provides a better objective response for both types of relapse, a similar OS for sensitive-relapsed cases, better OS for refractory-relapsed cases, and a similar AE profile except for a higher risk febrile neutropenia. AMR seemed much more beneficial for Japanese patients (Table 2, Supplementary Table 2). However, compared to the efficacy of TOP presented in the previous meta-analysis, MAR may be a better treatment option than TOP for both Japanese and Euro-American. Further phase III trials to compare AMR and TOP focusing on refractory-relapsed SCLC is anticipated.

## Additional Information

**How to cite this article**: Horita, N. *et al.* Amrubicin for relapsed small-cell lung cancer: a systematic review and meta-analysis of 803 patients. *Sci. Rep.* **6**, 18999; doi: 10.1038/srep18999 (2016).

## Supplementary Material

Supplementary Information

## Figures and Tables

**Table 1:** List of included studies.

Study	Country	Design	Phase	N	Dosage mg/m^2^	PS 0/1/2	Median age	Male (%)	Relapse type cutoff	Refractory relapse (%)	Third line (%)
Ettinger 2010^8^	USA	Single arm AMR	II	75	40	24/38/13	63	48	90 days	100	0
Igawa 2007^9^	Japan	Single arm AMR	?	27	40(a)	2/21/4	64	85	8 weeks	30	52
Inoue 2008^10^	Japan	RCT, vs TOP	II	29	40	14/10/5	70	83	90 days	41	0
Inoue 2015^11^	Japan	RCT, vs re-challenge	II	27	40	15/10/2	64	96	90 days	0	0
Jotte 2011^12^	USA	RCT, vs TOP	II	50	40	20/24/6	63	46	90 days	0	0
Kaira 2010^13^	Japan	Single arm AMR(b)	II	29	35	12/12/5	67	86	90 days	66	24
Murakami 2014^14^	Japan	Single arm AMR	II	82	40	34/48/0	66	79	90 days	100	12
Onoda 2006^15^	Japan	Single arm AMR	II	60	40	28/28/4	67	77	60 days	27	23
Pawel 2014^16^	Germany	RCT, vs TOP	III	424	40	126/289/9	62	58	90 days	47	0

RCT: randomized controlled trial. AMR: amrubicin. TOP: topotecan. N: number of small cell lung carcinoma (SCLC) patients treated with amrubicin. PS: performance status.

(a): Igawa *et al.* used 40 mg/m^2^ for all of the second-line cases (N = 20) and a half of the third-line cases (N = 7), but the other half of the third-line cases (N = 7) were treated with 35 mg/m^2^.

(b) Kaira *et al.* evaluated both SCLC and non-SCLC. We extracted only data concerning SCLC.

**Table 2:** Estimated responses and survivals for four second-line scenarios.

	Sensitive-R Japanese	Refractory-R Japanese	Sensitive-R Euro-American	Refractory-R Euro-American
Response rate	61% (50–71%)	38% (28–47%)	43% (36–49%)	19% (13–26%)
Disease control rate	83% (73–93%)	82% (72–91%)	69% (62–76%)	68% (61–75%)
3 m progression-free survival	72% (59–85%)	58% (42–74%)	66% (55–77%)	52% (42–64%)
6 m progression-free survival	36% (20–52%)	15% (0–34%)	40% (25–55%)	18% (4–32%)
9 m progression-free survival	16% (5–28%)	7% (0–19%)	16% (7–25%)	7% (0–15%)
6 m overall survival	83% (70–96%)	74% (61–88%)	62% (50–74%)	53% (42–65%)
12 m overall survival	51% (35–66%)	34% (18–50%)	35% (21–50%)	19% (5–33%)
18 m overall survival	24% (7–42%)	15% (0–33%)	15% (0–31%)	5% (0–21%)

m, month. Sensitive-R, sensitive-relapsed. Refractory-R, refractory-relapsed.

One cell indicates a result from one meta-regression. The estimated rate of response or survival accompanied with 95% CI was presented. Three co-variables were used for each analysis: sensitive relapse compared to refractory relapse, the Japanese study compared to the Euro-American study, and the second-line chemotherapy compared to the third-line chemotherapy.
