# Supplementary material for: Amrubicin for relapsed small-cell lung cancer: a systematic review and meta-analysis of 803 patients
Source: Sci Rep. 2016 Jan 11;6:18999. doi: 10.1038/srep18999 (PMC4707435; doi:10.1038/srep18999)

**Amrubicin for relapsed small-cell lung cancer: a systematic review and meta-analysis of 803 patients**

**Supplementary file**

**Authors**

^1)^Nobuyuki Horita, ^1)^Masaki Yamamoto, ^1)^Takashi Sato, ^1)^Toshinori Tsukahara, ^1)^Hideyuki Nagakura, ^1)^Ken Tashiro, ^1)^Yuji Shibata, ^1)^Hiroki Watanabe, ^1)^Kenjiro Nagai, ^1)^Kentaro Nakashima, ^1)^Ryota Ushio, ^1)^Misako Ikeda, ^2)^Nobuaki Kobayashi, ^3)^Masaharu Shinkai, ^3)^Makoto Kudo, ^1)^Takeshi Kaneko.

^1)^Department of Pulmonology, Yokohama City University Graduate School of Medicine, Yokohama, Japan.

^2)^Department of Pulmonology, Yokohama Minami Kyosai Hospital, Yokohama, Japan.

^3)^Respiratory Disease Center, Yokohama City University Medical Center, Yokohama, Japan.

Supplementary Table 1. Raw data concerning responses, survivals and adverse effects.

| Study | RR | DCR | 3m PFS | 6m PFS | 12m PFS | 6m OS | 12m OS | 18m OS |
| --- | --- | --- | --- | --- | --- | --- | --- | --- |
| Ettinger 2010 | 16/75 | 46/75 | 41/75 | 13/75 | 5/75 | 37/75 | 13/75 | 2/75 |
| Igawa 2007 | 8/27 | 16/27 | / | / | / | 19/27 | 9/27 | 3/27 |
| Inoue 2008 | 11/29 | 23/29 | 17/29 | 4/29 | 1/29 | 18/29 | 7/29 | 0/29 |
| Inoue 2015 | 18/27 | 22/27 | 22/27 | 13/27 | 7/27 | 24/27 | 16/27 | 10/27 |
| Jotte 2011 | 22/50 | 33/50 | 29/50 | 19/50 | 9/50 | 30/50 | 18/50 | 5/50 |
| Kaira 2010 | 13/29 | 23/29 | 22/29 | 8/29 | 1/29 | 20/29 | 15/29 | 5/29 |
| Murakami 2014 | 27/82 | 64/82 | 49/82 | 21/82 | 8/82 | 65/82 | 29/82 | 16/82 |
| Onoda 2006 | 31/60 | 43/60 | / | / | / | 50/60 | 30/60 | 17/60 |
| Pawel 2014 | 132/424 | 297/424 | 263/424 | 134/424 | 45/424 | 263/424 | 121/424 | 68/424 |
| Study | Neutro-  penia | Thrombo-  cytopenia | Anemia | FN | NV | Fatigue | Cardio-  toxicity | TRD |
| Ettinger 2010 | 46/69 | 28/69 | 21/69 | 8/69 | 6/69 | 15/69 | 2/69 | 3/69 |
| Igawa 2007 | 16/27 | 8/27 | 2/27 | 4/27 | 0/27 | 1/27 | 0/27 | 0/27 |
| Inoue 2008 | 27/29 | 8/29 | 6/29 | 4/29 | 1/29 | 5/29 | 0/29 | 1/29 |
| Inoue 2015 | 24/27 | 4/27 | 4/27 | 5/27 | 0/27 | 0/27 | 0/27 | 0/27 |
| Jotte 2011 | 30/49 | 19/49 | 12/49 | 5/49 | 0/49 | 6/49 | 1/49 | 4/49 |
| Kaira 2010 | 12/29 | 1/29 | 0/29 | 0/29 | 0/29 | 0/29 | 0/29 | 0/29 |
| Murakami 2014 | 77/82 | 17/82 | 21/82 | 22/82 | 1/82 | 1/82 | 0/82 | 0/82 |
| Onoda 2006 | 50/60 | 12/60 | 20/60 | 3/60 | 3/60 | 0/60 | 1/60 | 0/60 |
| Pawel 2014 | 169/408 | 86/408 | 65/408 | 41/408 | / | 43/408 | 21/408 | / |

RR: response rate. DCR: disease control rate. m: month. PFS: progression-free survival. OS: overall survival. FN: febrile neutropenia. NV: nausea vomiting. TRD: treatment-related death

Cases with response/survival/adverse events were presented as numerator. Total number of patients was presented as denominator. Total numbers of patients for response/survival and adverse effect are not always consistent because patients who did not receive amrubicin were excluded for adverse effect analysis.

Supplementary Table 2. Meta-regression for response and survival analysis: partial correlation coefficient by modulators.

|  | Sensitive vs refractory | | Japanese vs Euro-American | |
| --- | --- | --- | --- | --- |
|  | PCC (95% CI) | *p* | PCC (95% CI) | *p* |
| Response rate | 23% (12-34%) | <0.001 | 18% (9-27%) | <0.001 |
| Disease control rate | 1% ((-10)-12%) | 0.845 | 14% (5-23%) | 0.002 |
| 3 m progression-free survival | 14% ((-8)-20%) | 0.093 | 6% ((-8)-20%) | 0.414 |
| 6 m progression-free survival | 22% (1-42%) | 0.039 | -4% ((-21)-14%) | 0.675 |
| 9 m progression-free survival | 9% ((-4)-22%) | 0.164 | 1% ((-11)-12%) | 0.914 |
| 6 m overall survival | 9% ((-7)-25%) | 0.260 | 21% (7-35%) | 0.003 |
| 12 m overall survival | 17% ((-2)-35%) | 0.085 | 15% ((-1)-32%) | 0.073 |
| 18 m overall survival | 10% ((-12)-31%) | 0.384 | 10% ((-9)-29%) | 0.297 |

m, month. PCC, partial correlation coefficient. 95% CI: 95% confidence interval.

One line indicates a result from one meta-regression. Three co-variables were used for each analysis: sensitive relapse compared to refractory relapse, the Japanese study compared to the Euro-American study, and the second-line chemotherapy compared to the third-line chemotherapy.

For example, the response rate was 23% higher among sensitive relapse cases compared to refractory relapse cases, and 18% higher in Japanese studies compared to Euro-American studies. Table 2 may help further understanding of this table.

For each meta-regression, the outcome presented in the left row was used

Supplementary Figure 1. Additional forest plots for objective responses, survivals, and adverse effects.

SE: standard error. IV: inverse variance method. 95% CI: 95% confidence interval.


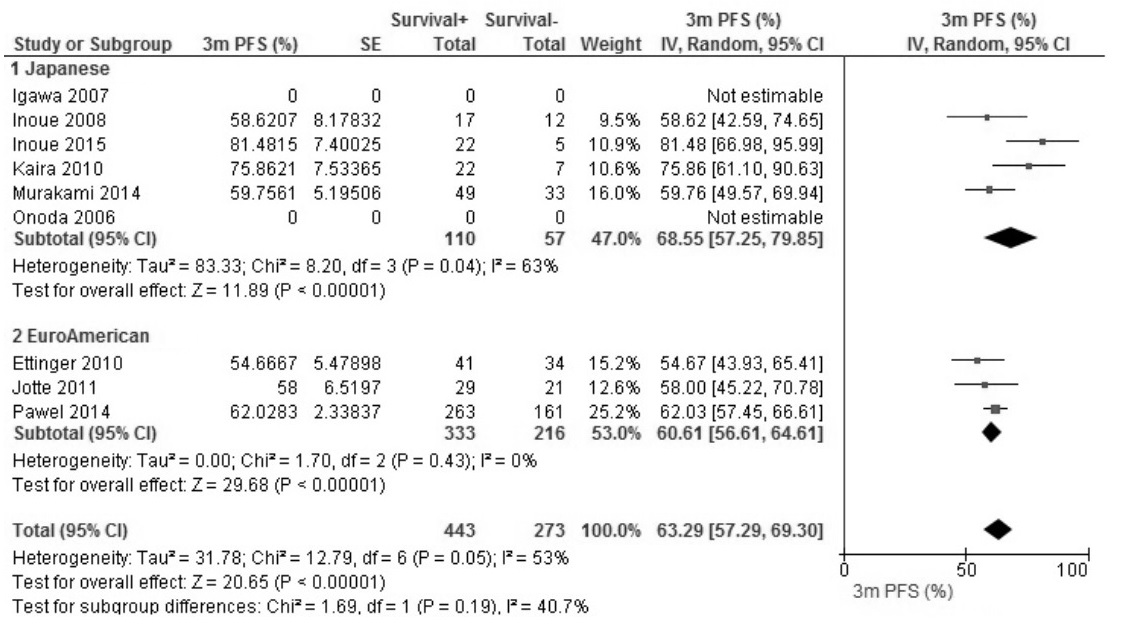
(a) 3-month progression-free survival (3m PFS)

(b) 9-month progression-free survival (9m PFS)


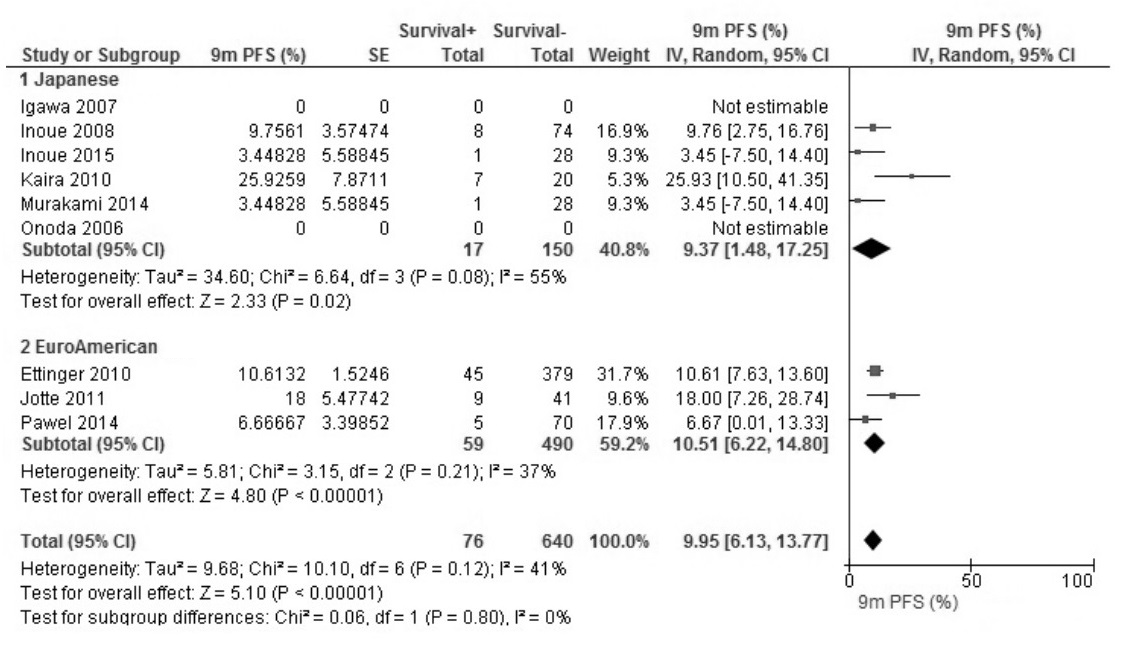


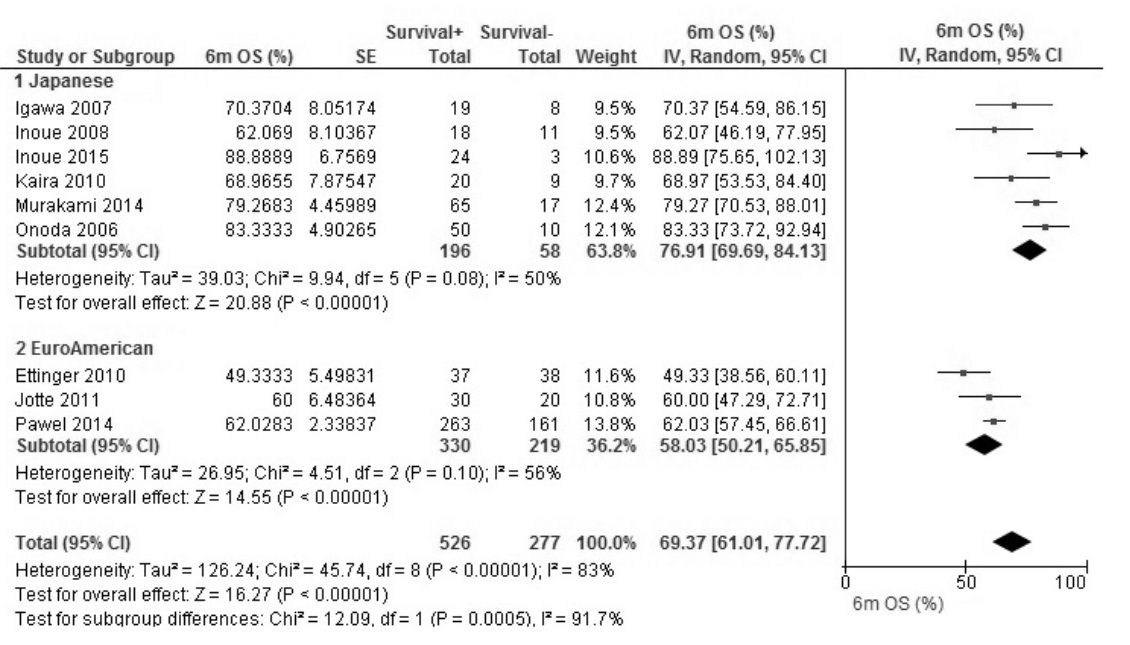
(c) 6-month overall survival (6m OS)

(d) 18-month overall survival (18m OS)


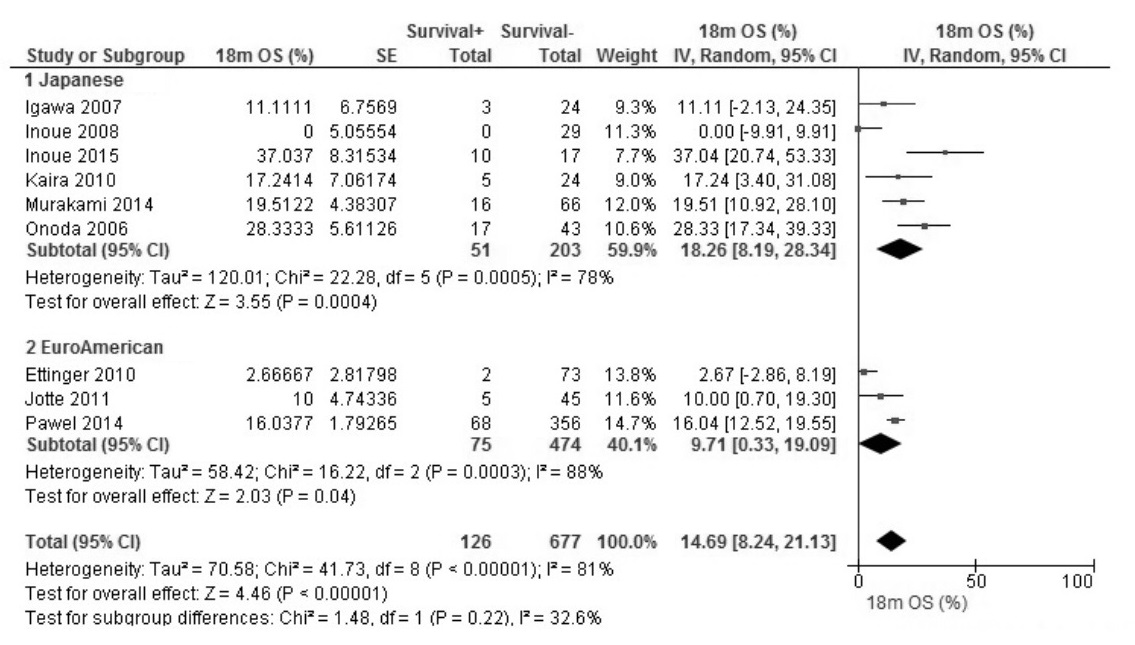


(e) Thrombopenia


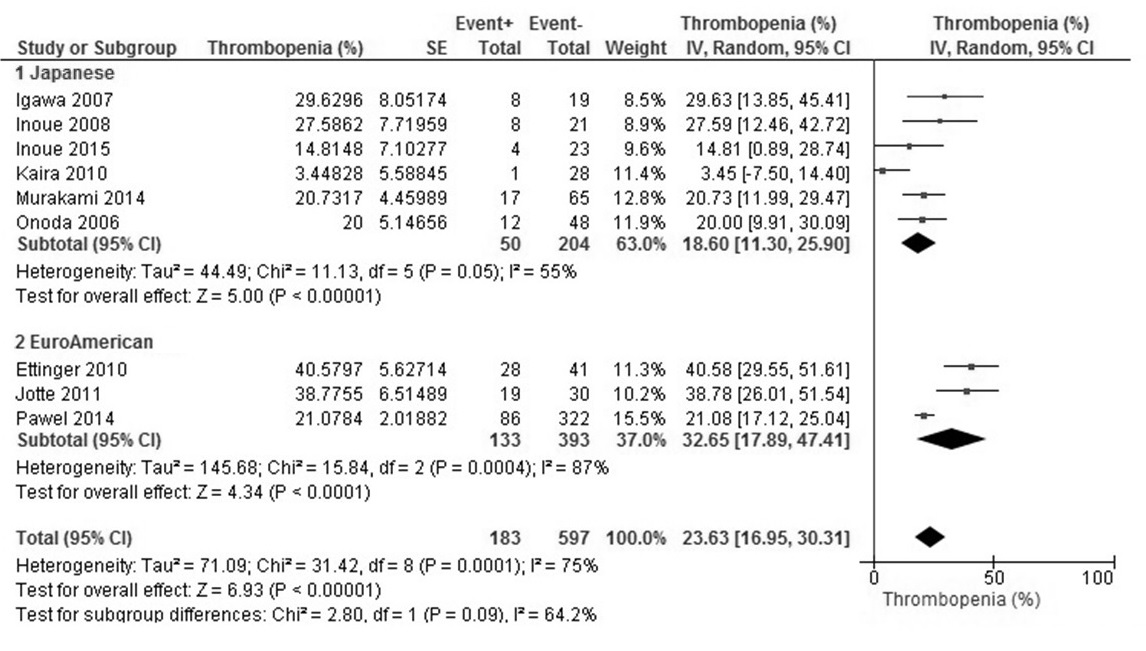


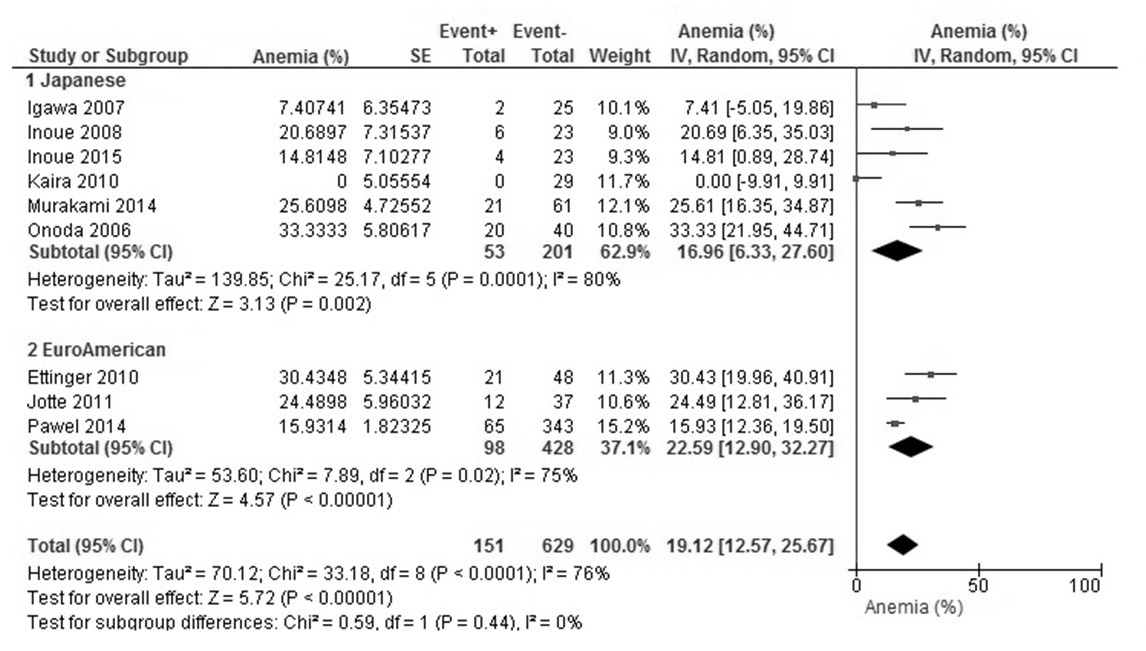
(f) Anemia


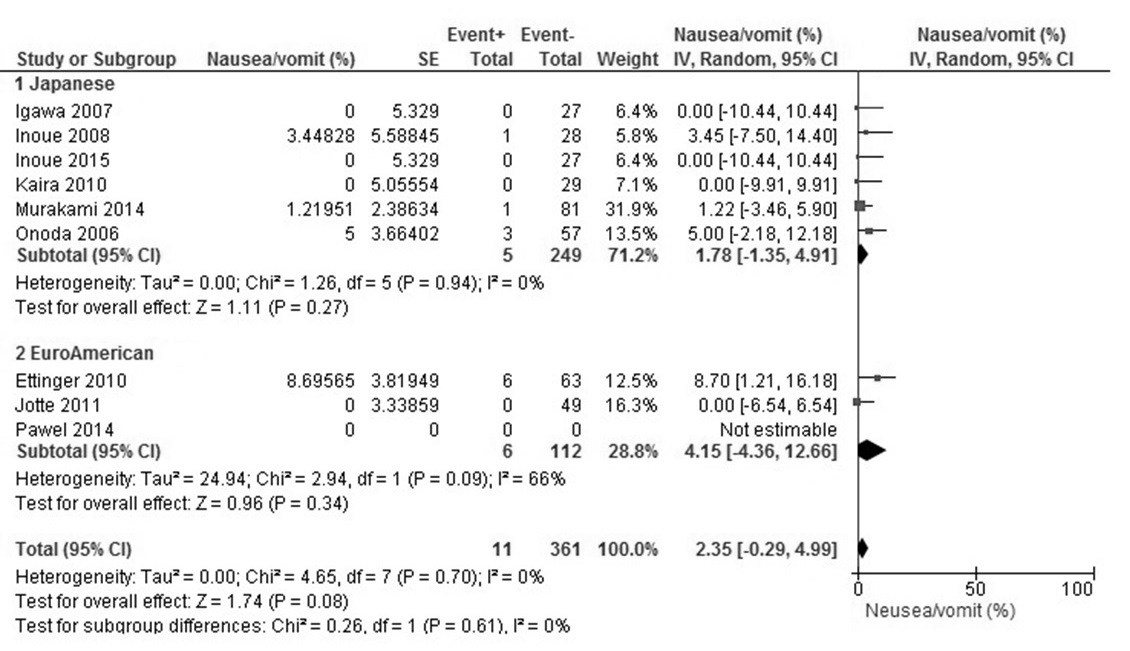
(g) Nausea/vomiting

(h) Fatigue


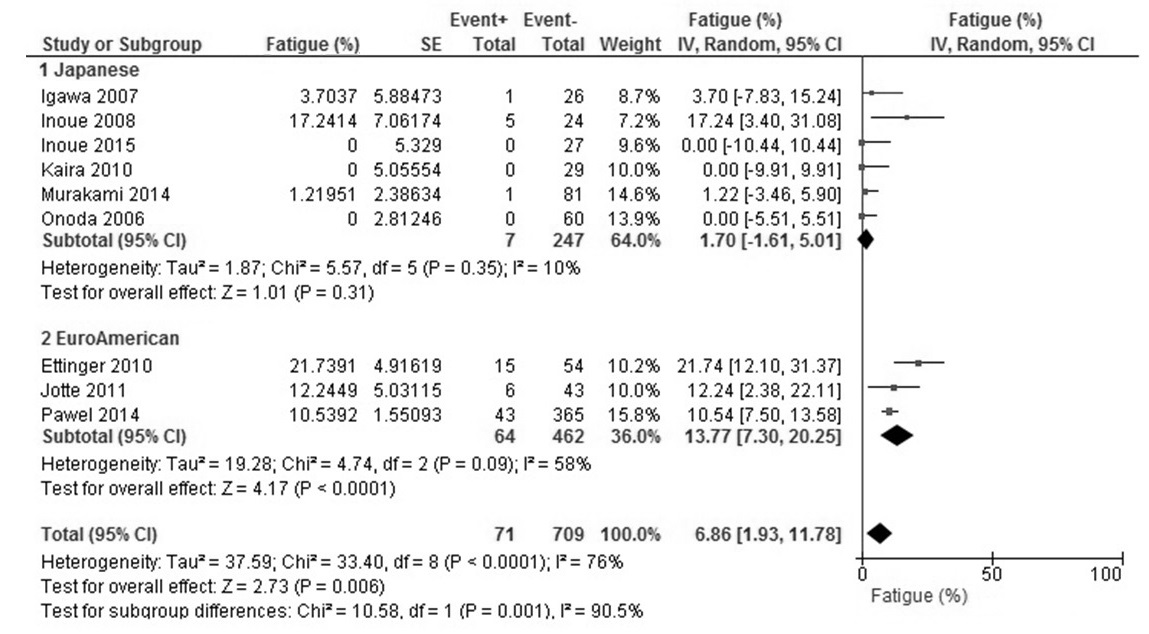


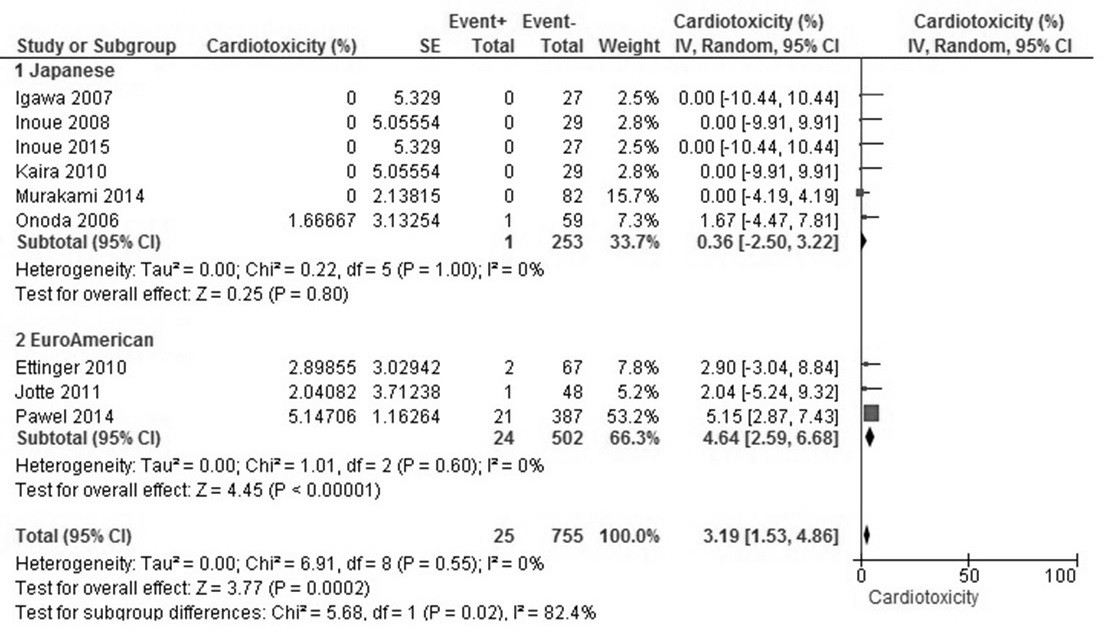
(i) Cardiotoxicity


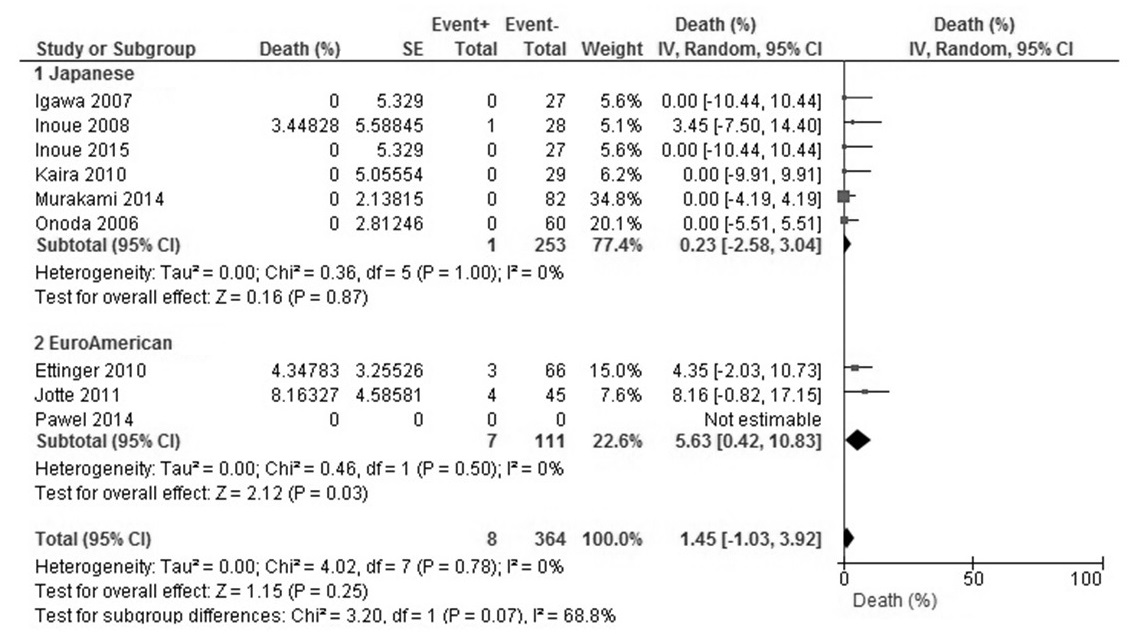
(j) Treatment-related death

Supplementary Figure 2. Funnel plots for objective responses, survivals, and adverse effects.

SE: standard error.

Small SE usually suggests that the study was supported by a large sample size and a large number of events.

No statistical test was conducted for publication bias because number of trials did not exceed 10. Visual inspection of funnel plots revealed no publication bias for any of the meta-analyses.

1. Response rate (RR).


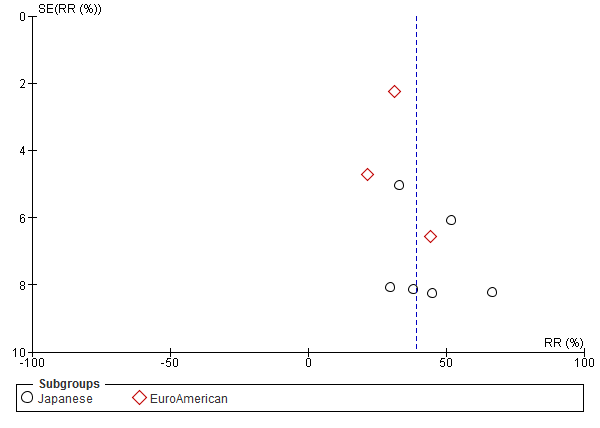


(b) Disease control rate (DCR).


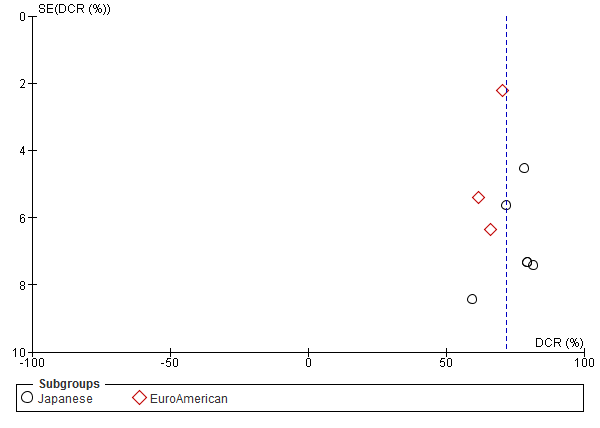


(c) Three-month progression-free survival (3m PFS).


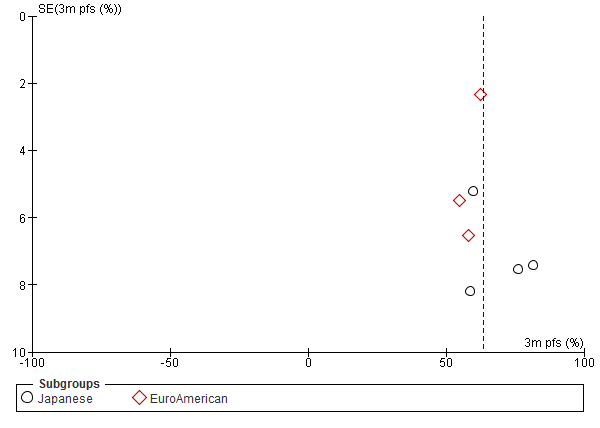


(d) Six-month progression-free survival (6m PFS).


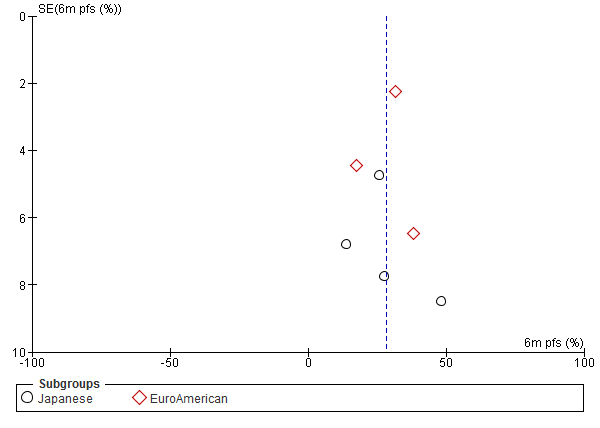


(e) Nine-month progression-free survival (9m PFS).


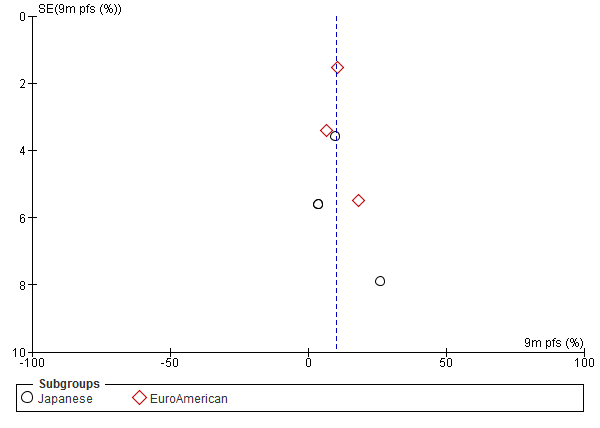


(f) Six-month overall survival (6m OS).


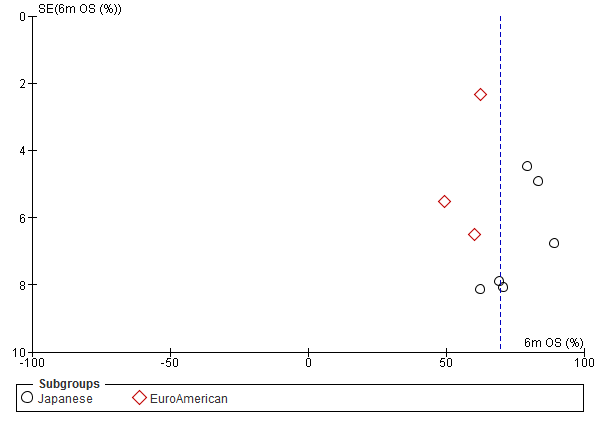


(g) Twelve-month overall survival (12m OS).


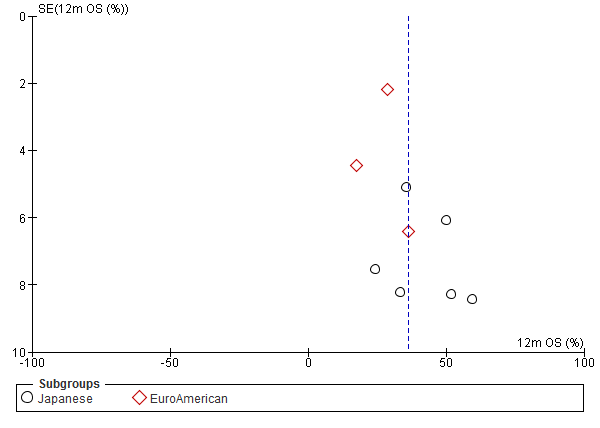


(h) Eighteen-month overall survival (18m OS).


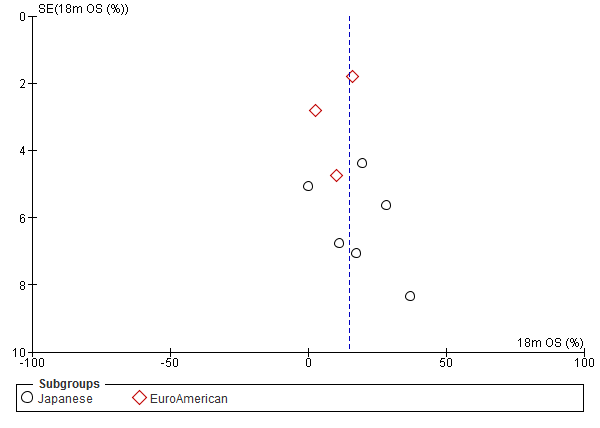


(i) Neutropenia


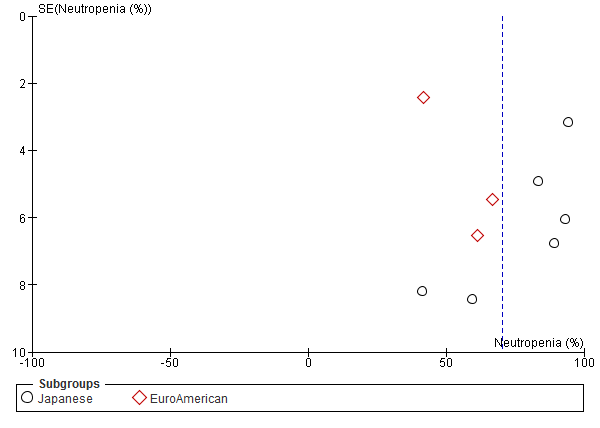


(j) Thrombopenia


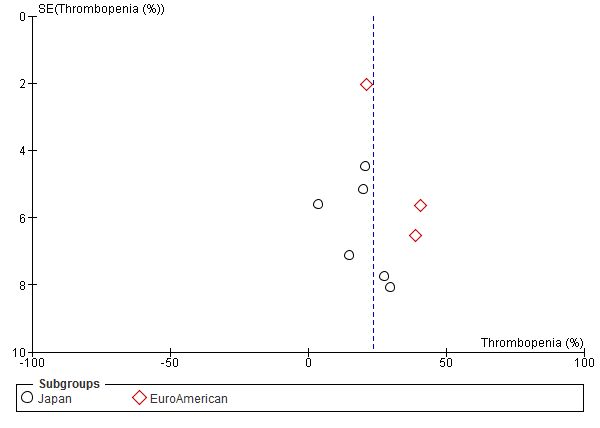


(k) Anemia


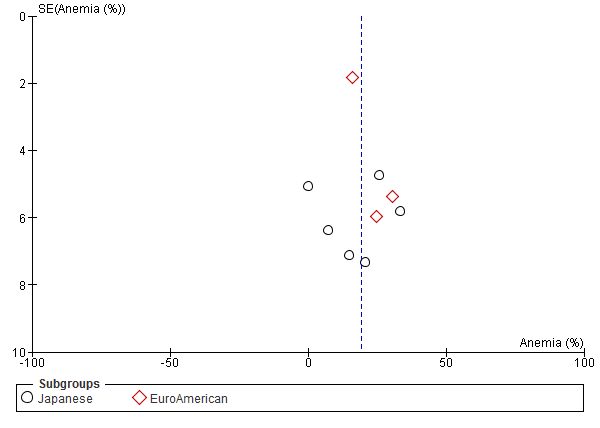


(l) Febrile (Fbl) neutropenia.


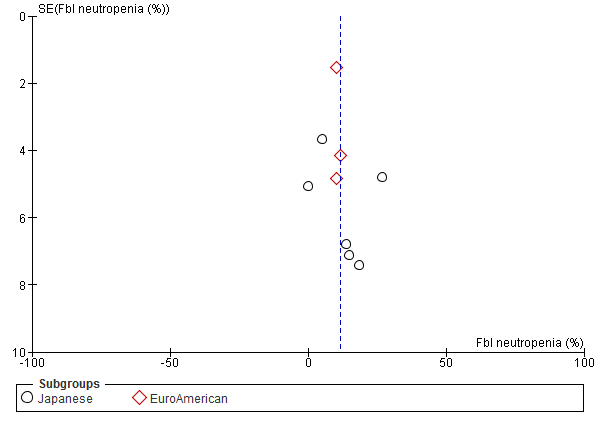


(m) Nausea/vomiting


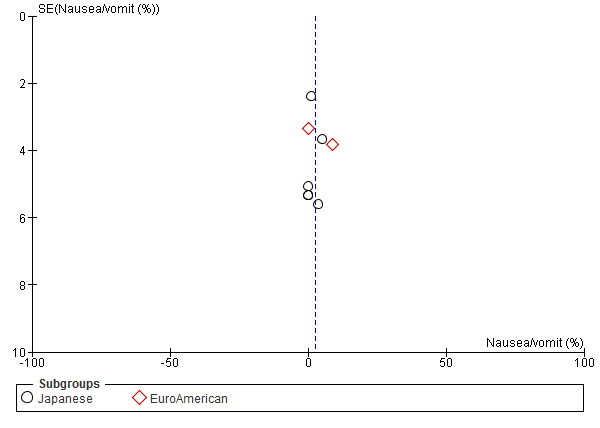


(n) Fatigue


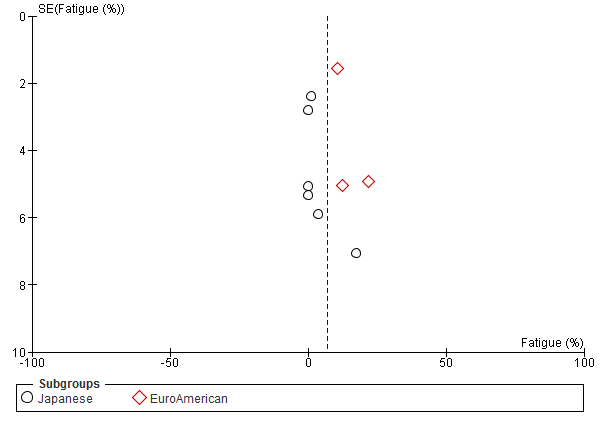


(o) Cardiotoxicity


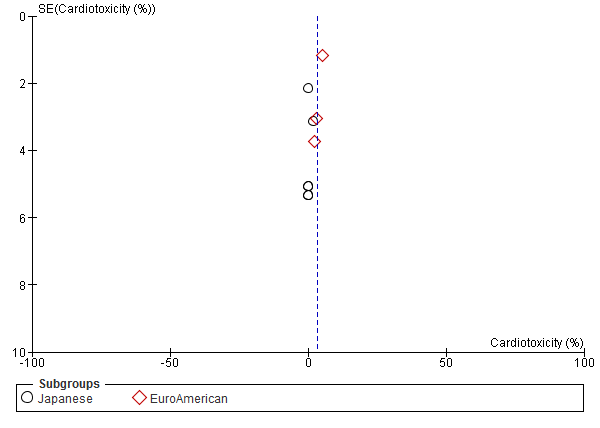


(p) Treatment-related death


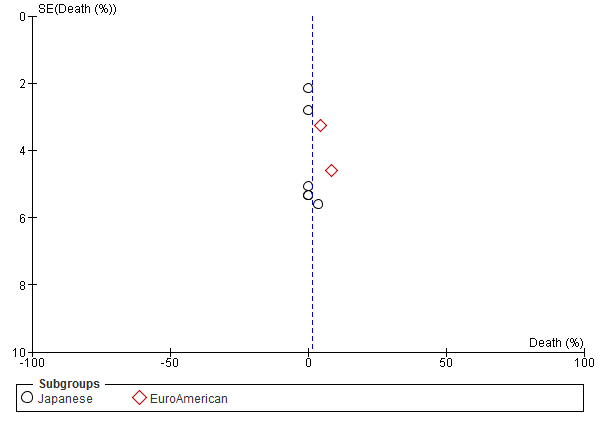

Supplement: Supplementary Information [file srep18999-s1.doc]
